# Impact of five annual rounds of mass drug administration with ivermectin on onchocerciasis in Sierra Leone

**DOI:** 10.1186/s40249-018-0410-y

**Published:** 2018-04-06

**Authors:** Joseph B. Koroma, Santigie Sesay, Abdul Conteh, Benjamin Koudou, Jusufu Paye, Mohamed Bah, Mustapha Sonnie, Mary H. Hodges, Yaobi Zhang, Moses J. Bockarie

**Affiliations:** 1 Family Health International (FHI) 360, Ghana Country Office, Accra, Ghana; 2 National Neglected Tropical Disease Control Programme, Ministry of Health and Sanitation, Freetown, Sierra Leone; 3 Centre for Neglected Tropical Diseases, Liverpool School of Tropical Medicine, Liverpool, UK; 4 Helen Keller International, Freetown, Sierra Leone; 5 Helen Keller International, Regional Office for Africa, Dakar, Senegal; 6 European & Developing Countries Clinical Trials Partnership (EDCTP), Medical Research Council, Cape Town, South Africa

**Keywords:** Onchocerciasis, *Onchocerca volvulus*, Mass drug administration, Community-directed treatment with ivermectin, Community-directed drug distributor, Skin snip, Onchocerciasis-endemic, Post-conflict, Disease elimination, Rapid diagnostic test

## Abstract

**Background:**

Onchocerciasis is endemic in 12 of the 14 health districts of Sierra Leone. Good treatment coverage of community-directed treatment with ivermectin was achieved between 2005 and 2009 after the 11-year civil conflict. Sentinel site surveys were conducted in 2010 to evaluate the impact of five annual rounds of ivermectin distribution.

**Methods:**

In total, 39 sentinel villages from hyper- and meso-endemic areas across the 12 endemic districts were surveyed using skin snips in 2010. Results were analyzed and compared with the baseline data from the same 39 villages.

**Results:**

The average microfilaridermia (MF) prevalence across 39 sentinel villages was 53.10% at baseline. The MF prevalence was higher in older age groups, with the lowest in the age group of 1–9 years (11.00%) and the highest in the age group of 40–49 years (82.31%). Overall mean MF density among the positives was 28.87 microfilariae (mf)/snip, increasing with age with the lowest in the age group of 1–9 years and the highest in the age group of 40–49 years. Males had higher MF prevalence and density than females. In 2010 after five rounds of mass drug administration, the overall MF prevalence decreased by 60.26% from 53.10% to 21.10%; the overall mean MF density among the positives decreased by 71.29% from 28.87 mf/snip to 8.29 mf/snip; and the overall mean MF density among all persons examined decreased by 88.58% from 15.33 mf/snip to 1.75 mf/snip. Ten of 12 endemic districts had > 50% reduction in MF prevalence. Eleven of 12 districts had ≥50% reduction in mean MF density among the positives.

**Conclusions:**

A significant reduction of onchocerciasis MF prevalence and mean density was recorded in all 12 districts of Sierra Leone after five annual MDAs with effective treatment coverage. The results suggested that the onchocerciasis elimination programme in Sierra Leone was on course to reach the objective of eliminating onchocerciasis in the country by the year 2025. Annual MDA with ivermectin should continue in all 12 districts and further evaluations are needed across the country to assist the NTDP with programme decision making.

**Electronic supplementary material:**

The online version of this article (10.1186/s40249-018-0410-y) contains supplementary material, which is available to authorized users.

## Multilingual abstracts

Please see Additional file 1 for translations of the abstract into the five official working languages of the United Nations.

## Background

Onchocerciasis, also known as river blindness or Robles’ disease, is a parasitic disease caused by infection with the parasite *Onchocerca volvulus*. The disease is transmitted to humans by the black fly (*Simulium spp.*) and its pathology is linked to the death of the microfilariae in the skin and eyes [1–3]. Humans are known as the main reservoirs for *O. volvulus* [4]. Some animals such as elands and buffalos are possible reservoir hosts, which makes control of the disease in areas where these animals co-exist more difficult [5]. Currently, there are an estimated 187 million people at risk of onchocerciasis among which 37 million are infected with *O. volvulus*. Among those infected, an estimated 4 million people live with skin manifestations of the disease and 2 million are estimated to be either visually impaired or blind [1–4]. Onchocerciasis is the world’s second leading infectious cause of blindness after trachoma. Blindness from onchocerciasis occurs early in life (20–30 years old) and therefore creates socio-economic problems for those affected, their families and their communities [1–4]. About 99% of reported cases of the disease are in 31 endemic countries in sub-Saharan Africa [1, 3, 6–8].

Onchocerciasis control in Africa started with the launching of the Onchocerciasis Control Programme in West Africa (OCP) in seven countries (Benin, Burkina Faso, Cote d’Ivoire, Ghana, Mali, Niger and Togo) in 1974 focusing on vector control and then continued with the African Programme for Onchocerciasis Control (APOC) in 1995 focusing on community-directed treatment with ivermectin (CDTI) in meso- (microfilaridermia [MF] prevalence 40%–59.9%) and hyper- (MF prevalence ≥60%) endemic sites [9]. Since 2009 there has been a paradigm shift from control of onchocerciasis as a public health problem (reduction of *O. volvulus* MF prevalence to an acceptable low level where transmission may continue) to elimination of the disease by stopping local transmission. This shift was motivated by studies in Senegal and Mali which demonstrated that through treatment with ivermectin it was possible to eliminate the disease [10]. It is currently believed that below 5% *O. volvulus* MF prevalence the disease prevalence would continue to drop even in the absence of treatment and transmission would stop eventually [4, 10]. The recommended programme coverage (i.e. proportion of people ingesting ivermectin among people targeted in the endemic districts that are eligible for treatment, i.e. ≥5 years) during treatment with ivermectin is ≥80% in all endemic areas [3, 4, 10–13].

**Fig. 1:**
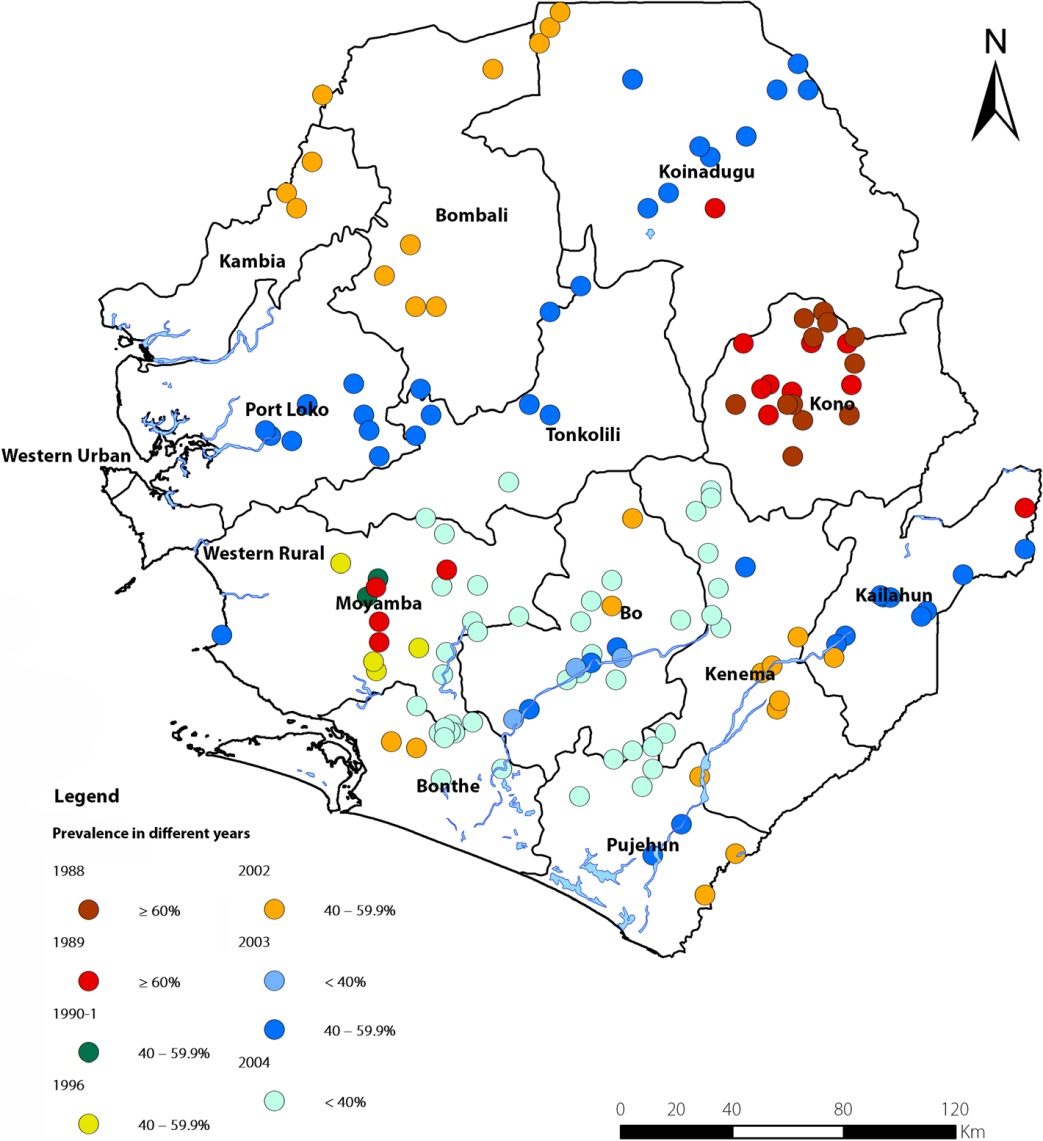
Distribution map of unadjusted point prevalence of onchocerciasis from baseline surveys in Sierra Leone. Data were collected during 1988 and 2004. Prevalence results from different years are plotted separately

The endemicity of onchocerciasis was demonstrated in Sierra Leone in 1926 when Blacklock first described its transmission through the black fly (*S. damnosum*) in the Kono district [14]. Onchocerciasis control efforts in the country started as early as in 1957 with insecticide treatments along the Tonkolili River that was found to be the most severely affected [15]. It was documented that onchocerciasis was the second most common cause of blindness after cataracts in Sierra Leone and in the late 1980s the former OCP extended its activities to four other countries - Guinea, Guinea-Bissau, Senegal and Sierra Leone [16–19]. Considerable work since the 1950s demonstrated high onchocerciasis prevalence in Sierra Leone along the main rivers and existence of black flies in the entire country except in areas around the capital of Freetown and the southern coastal plain of the Bonthe district [20]. During 1988–2004, surveys from 177 sites across all 14 districts, selected based on proximity to rivers and surveyed using the skin snip method, showed that the unadjusted MF prevalence varied from 0% to 78.3% (see Fig. 1) [20]. With technical assistance from APOC, villages around survey sites were classified to be hypo-, meso- or hyper-endemic according to the adjusted MF prevalence. The historical data and timelines of onchocerciasis control activities in Sierra Leone is summarized in Table 1.

**Table 1:** Historical data and time lines of onchocerciasis control activities in Sierra Leone

Year	Event	Comments
1926	The endemicity of onchocerciasis was demonstrated in Sierra Leone when Blacklock first described its transmission through the black fly, *S. damnosum* in the Kono district	
1957	Start of onchocerciasis control efforts with insecticide treatment along the Tonkolili River that was found to be the most severely affected	
1974	Launch of the Onchocerciasis Control Programme in West Africa (OCP) in 7 countries (Benin, Burkina Faso, Cote d’Ivoire, Ghana, Mali, Niger and Togo)	Focus was on vector control but chemotherapy was added in the late 1980s and early 2000s
1988/9	OCP was extended to four other countries - Guinea, Guinea-Bissau, Senegal and Sierra Leone	
1989	The National Onchocerciasis Control Program (NOCP) was established in Sierra Leone under the OCP	
1995	Launch of the African Programme for Onchocerciasis Control (APOC)	Initiated the community-directed treatment with ivermectin (CDTI) strategy
1991–2002	Civil conflict in Sierra Leone resulted in limited onchocerciasis activities	Treatment coverage before 2002 are considered unreliable
2003	Onchocerciasis control activities was restarted as part of the Special Intervention Zones (SIZ) programme (2003–2007) that was managed by APOC	CDTI was implemented in meso- and hyper-endemic areas of the 12 endemic districts with very poor epidemiological and geographic coverage in 2003–2004
1998–2005	Epidemiological mapping was conducted with support from APOC, showing high onchocerciasis prevalence along the main rivers and existence of black flies in the entire country except in areas around the capital Freetown and the southern coastal plain of the Bonthe district	A total of 177 sites were surveyed using skin snip method across 14 districts in the country.
2005	Management changes made within the NOCP by the Ministry of Health and Sanitation (MOHS) to improve onchocerciasis control efforts in the country	Better treatment coverage reported since then
2007	The NOCP expanded to become the national integrated Neglected Tropical Disease Programme (NTDP) to include onchocerciasis, lymphatic filariasis (LF), schistosomiasis and soil-transmitted helminths. Trachoma was demonstrated to be non-endemic in Sierra Leone.	Onchocerciasis and LF activities integrated since then in all 12 co-endemic districts by co-administering albendazole and ivermectin treating all hyper-, meso- and hypo-endemic villages for onchocerciasis.
2009	Paradigm shift from control of onchocerciasis as a public health problem (reduction of *O. volvulus* microfilaridermia prevalence to an acceptable low level although transmission will continue) to eliminating the disease by stopping local transmission	This was after studies in Senegal and Mali showed that through treatment with ivermectin it is possible to eliminate the disease

The National Onchocerciasis Control Programme (NOCP) in Sierra Leone was established in 1989 under the OCP. However, the civil conflict between 1991 and 2002 negatively impacted on onchocerciasis control activities, and from 1997 to 2002 only limited onchocerciasis control activities were conducted in areas with high prevalence and with security; therefore, treatment coverage before 2002 was deemed not reliable. With financial and technical support from APOC, the NOCP restarted interventions in 2003 through the Special Intervention Zones (SIZ) programme (2003–2007). CDTI was implemented in meso- and hyper-endemic areas in the 12 endemic districts [17–19, 21]. However, epidemiological coverage (i.e. proportion of people ingesting ivermectin among the total population in the targeted endemic districts) was reported to be 36% and 28% in 2003 and 2004 respectively, and geographic coverage (i.e. proportion of endemic communities and districts targeted among all the endemic communities and districts needing treatment) could not be determined for the two years [20]. Efforts to control onchocerciasis improved in 2005, in part due to changes effected within the programme management by the Ministry of Health and Sanitation (MOHS) to improve onchocerciasis control efforts in the country [20]. Treatment coverage has significantly improved since 2005.

In 2007, the NOCP was expanded to become the national integrated Neglected Tropical Disease Programme (NTDP) to include onchocerciasis, lymphatic filariasis (LF), schistosomiasis and soil-transmitted helminths. LF was endemic in all 14 districts and co-endemic with onchocerciasis in 12 districts. Therefore, co-administering albendazole and ivermectin in all LF endemic districts effectively treated all hyper-, meso- and hypo-endemic villages for onchocerciasis.

The current World Health Organisation (WHO) guidelines on onchocerciasis elimination recommend impact assessment following 3–5 annual rounds of effective ivermectin distribution [22]. In line with these recommendations, a national sentinel site assessment was conducted in 2010–2011 using the skin snip method. The purpose of this paper is to determine the impact of five annual rounds of ivermectin distribution on the transmission of onchocerciasis. We compared the data from sentinel sites across the 12 onchocerciasis-endemic districts at baseline and in 2010–2011 and discussed the way forward in reaching onchocerciasis elimination in Sierra Leone.

## Methods

### Mass drug administration

Annual CDTI started in 2003. However, effective treatment coverage was not achieved until 2005 due to uncertainty around population data post-war and the internal migration of displaced people. Integrated, annual onchocerciasis/LF mass drug administration (MDA) with ivermectin and albendazole was piloted in 2007 in six districts located in border areas with neighbouring Guinea and Liberia, while CDTI continued in the other six districts. Integrated onchocerciasis/LF MDA was scaled up to cover all 12 coendemic districts in 2008. Community-directed drug distributors (CDDs) used dose poles to distribute ivermectin and later ivermectin plus albendazole to the eligible population aged five years and older. The CDDs were literate members of communities that were selected by their communities. District health workers conducted training of CDDs for MDA and provided supervision during MDA. NTDP staff and members of the district health management teams (DHMTs) also supported training and supervision of the MDA.

Distribution of medicines was recorded using community registers. The register was designed to capture all members of each community targeted for treatment. Before each MDA, CDDs conducted pre-MDA census and updated the community register to reflect those that had left the community, those that had joined the community and the newly born. MDA details were captured in the registers. After each MDA, the details were summarised on village reporting forms by CDDs and submitted to the supervising district health workers, who in turn summarised and submitted the reporting forms to include all villages they covered to DHMTs. Each DHMT then submitted the district summary report to the NTDP that collated results from all districts.

### Baseline surveys and sentinel sites

Baseline data were collected during 1988–2004 from 177 sites as illustrated in Fig. 1. The sites were randomly selected from all 14 districts of Sierra Leone based on their closeness to the ten major rivers and their tributaries [20]. To monitor the impact on the onchocerciasis prevalence and MF density after five rounds of MDA, 46 sites with high prevalence (MF prevalence ≥40%) from the 177 baseline sites were selected as sentinel sites. Seven of the 46 villages could not be located through field visits and had to be dropped from the list. The disappearance of villages was not unusual in the post-war context as much displacement and migration had occurred during the war. Accordingly, 39 sites (38 sites with MF prevalence ≥40% and one site below 40%) in the hyper- and meso-endemic areas were finally selected as the sentinel sites and a cross-sectional survey was conducted in 2010–11.

### Study population and parasitological detection

*O. volvulus* infection was determined in convenience samples using the method of microscopic examination of skin snips for the presence of *O. volvulus* microfilariae according to WHO/APOC recommendations. Skin snips were done in all selected villages 11–12 months after the last MDA. All volunteers/participants aged one year or above (at baseline) or five years and above (2010 survey) in each site were eligible for inclusion in the study without discrimination on gender, social status, religion or ethnicity. They were asked for identification data, including name, age, sex, occupation, and number of years they were resident in the village. Two skin biopsies were obtained from the right and left iliac crests of each participant. A 2 mm Holth corneoscleral punch was used to obtain the two bloodless skin snip biopsies. The scleral punch was sterilised with sodium hypo-chlorite solution and distilled water and then autoclaved for 15 min after taking biopsies from each participant.

The samples were microscopically examined for the presence of *O. volvulus* microfilariae after incubation for 30 min in distilled water. Negative skin snip samples were then kept in saline solution for another 24 h and microscopically re-examined. The number of microfilariae was counted and expressed as the number of microfilariae per snip, and the results were recorded for each person examined. For quality control, experts recruited by OCP and later APOC that worked in the field with the NOCP survey teams examined all positive slides and 10% of the negative slides.

### Statistical analysis

Results were entered into MS Excel and analysed in SPSS (IBM, Version 19, USA). Prevalence and density of MF were calculated by district, sex and age group, and were compared with the baseline data from the same 39 sites. The 95% confidence intervals (*CI*s) for prevalence were calculated using the Wilson score method without continuity correction [23]. The arithmetic mean MF density of infection with 95% *CI* was calculated for the total population examined (both positives and negatives) and for the positives only. The Chi-squared test was used to compare the differences in prevalence, and the Kruskal-Wallis test was used to compare the differences in MF density. Treatment coverage was calculated according to the WHO guidelines and reported using two indicators: epidemiological coverage and programme coverage [24]. The total population used was the total number of people registered during the pre-MDA census CDDs conducted each year. The distribution map of the unadjusted point prevalence of the 39 villages used as sentinel sites were produced using ArcGIS software version 10.4 (ESRI, Redlands, US) [25, 26].

## Results

### MDA results 2005–2009

In 2005–2007, ivermectin was distributed annually to a total of 8451 villages that were meso- and hyper-endemic for onchocerciasis, though about 50% of these villages conducted integrated onchocerciasis/LF MDA with ivermectin and albendazole in 2007. Between 2008 and 2009 ivermectin together with albendazole was distributed annually in the 12 provincial districts for treatment of both LF and onchocerciasis. The treatment area included the 8451 meso- and hyper-endemic onchocerciasis villages that received prior CDTI as well as the hypo-endemic villages that had not been treated under CDTI. Geographic coverage for onchocerciasis treatment of meso- and hyper-endemic communities in these districts during this period was 100%. As described previously, treatment coverage in 2003 and 2004 was ineffective and is not shown here. Table 2 shows treatment coverage between 2005 and 2009. Except for 2005, treatment coverage in other years for all districts was above the minimum required threshold.

**Table 2:** Summary results of annual MDA carried out for onchocerciasis in Sierra Leone 2005–2009^a^

Districts	2005				2006				2007				2008				2009			
	Total pop	Eligible pop^b^	No. treated	Epi/Prog Coverage^c^	Total pop	Eligible pop	No. treated	Epi/Prog Coverage	Total pop	Eligible pop	No. treated	Epi/Prog Coverage	Total pop	Eligible pop	No. treated	Epi/Prog Coverage	Total pop	Eligible pop	No. treated	Epi/Prog Coverage
Bo	251 523	201 218	193 861	77.1/96.3	257 444	205 955	181 006	70.3/87.9	308 371	246 697	219 651	71.2/89	333 941	267 153	235 427	70.5/88.1	363 824	291 059	268 222	73.7/92.2
Bombali	246 383	197 106	170 035	69/86.3	252 183	201 746	178 195	70.7/88.3	260 993	208 794	197 105	75.5/94.4	282 856	226 285	209 378	74/92.5	395 820	316 656	303 593	76.7/95.9
Bonthe	49 533	39 626	32 347	65.3/81.6	50 699	40 559	40 367	79.6/99.5	56 038	44 830	43 493	77.6/97	90 108	72 086	59 373	65.9/82.4	95 125	76 100	70 821	74.5/93.1
Kailahun	223 499	178 800	91 874	41.1/51.4	228 761	183 009	167 859	73.4/91.7	233 489	186 791	177 950	76.2/95.3	285 580	228 464	210 281	73.6/92	249 476	199 581	193 931	77.7/97.2
Kambia	99 961	8,4967	84 914	84.9/99.9	102 314	86 967	85 622	83.7/98.5	105 985	84 788	82 802	78.1/97.7	128 253	102 602	95 393	74.4/93	125 875	100 700	84 244	66.9/83.7
Kenema	212 541	170 033	117 021	55.1/68.8	217 545	174 036	171 765	79/98.7	218 823	175 058	164 789	75.3/94.1	287 569	230 055	210 935	73.4/91.7	229 661	183 729	172 236	75/93.7
Koinadugu	138 800	111 040	85 785	61.8/77.3	142 068	113 654	101 258	71.3/89.1	139 827	111 862	105 067	75.1/93.9	191 971	153 577	141 749	73.8/92.3	196 270	157 016	142 405	72.6/90.7
Kono	173 782	139 026	108 005	62.1/77.7	177 873	142 298	131 556	74/92.5	172 851	138 281	135 745	78.5/98.2	176 463	141 170	127 063	72/90	124 506	99 605	93 520	75.1/93.9
Moyamba	188 327	150 661	101 658	54/67.5	192 760	154 208	140 934	73.1/91.4	200 854	160 683	149 242	74.3/92.9	211 392	169 114	162 675	77/96.2	202 934	162 347	156 181	77/96.2
Port Loko	168 432	143 167	139 170	82.6/97.2	172 397	137 918	134 917	78.3/97.8	204 840	163 872	157 423	76.9/96.1	159 189	127 351	106 236	66.7/83.4	219 115	175 292	156 077	71.2/89
Pujehun	150 508	120 406	91 277	60.6/75.8	154 051	123 241	109 061	70.8/88.5	197 682	158 146	145 323	73.5/91.9	214 550	171 640	155 988	72.7/90.9	232 784	186 227	180 047	77.3/96.7
Tonkolili	236 038	188 831	101 379	43/53.7	241 595	193 276	184 953	76.6/95.7	252 833	202 266	192 167	76/95	258 792	207 034	177 206	68.5/85.6	340 078	272 062	256 268	75.4/94.2
TOTAL	2 139 327	1 724 882	1 317 326	61.6/76.4	2 189 690	1 756 868	1 627 493	74.3/92.6	2 352 586	1 882 069	1 770 757	75.3/94.1	2 620 664	2 096 531	1 891 704	72.2/90.2	2 775 468	2 220 374	2 077 545	74.9/93.6

Notes: ^a^ Geographic coverage of meso- and hyper-endemic onchocerciasis communities was 100% over the 5 years in all 12 districts. ^b^ Eligible population represents the population aged 5 years and over. ^c^ Epidemiological coverage represents the percentage proportion (%) of the persons treated among the total population and program coverage represents the percentage proportion (%) of the persons treated among the eligible population

### Onchocerciasis situation at baseline

#### MF prevalence

**Fig. 2:**
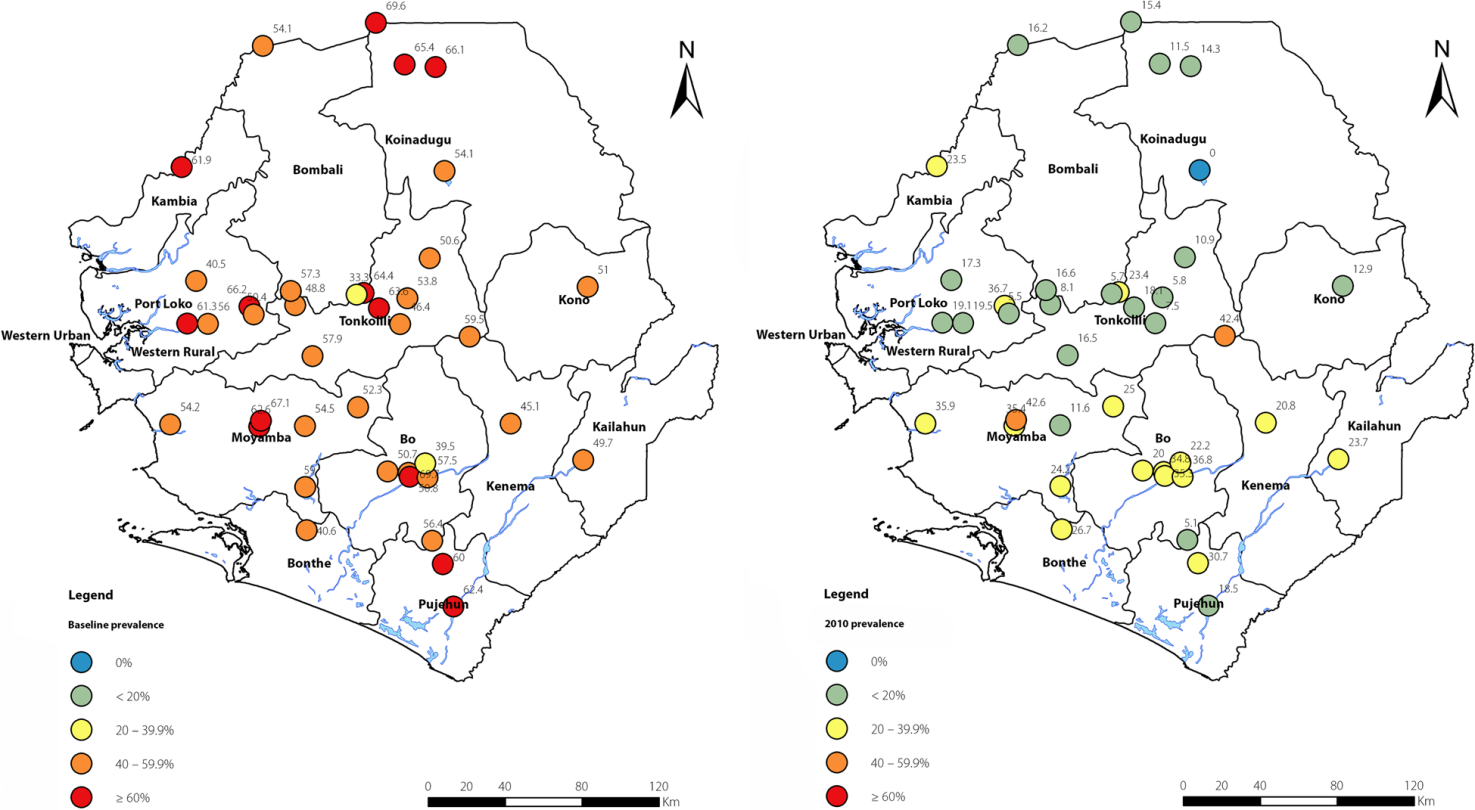
Unadjusted point prevalence of onchocerciasis at the 39 sentinel sites at baseline (1988–2004, on the left) and in 2010 (on the right). Numbers represent the actual precentage point MF prevalence at each sentinel site

The results of surveys at baseline are shown in Table 3. At baseline, a total of 7116 people were tested in all the 39 villages: 3461 (48.6%) males and 3655 (51.4%) females. Geographical locations of the 39 sentinel sites are shown in Fig. 2. The average baseline MF prevalence from the sentinel sites within each district varied from 39.01% (95% *CI*: 36.17–41.91%) to 61.94% (95% *CI*: 54.09–69.20%). MF prevalence in the northern districts (Kambia, Tonkolili, Koinadugu, Bombali and Port Loko) tended to be higher than prevalence in the southern districts (Moyamba, Bo, Pujehun and Bonthe), while the prevalence in the eastern districts was relatively the lowest (Kailahun, Kenema and Kono). The overall MF prevalence among all 7116 participants was 53.09% (95% *CI*: 51.93–54.25%). The MF prevalence was higher in males (55.19%, 95% *CI*: 53.52–56.84%) than in females (51.11%, 95% *CI*: 49.49–52.73%) (*P* < 0.001). Among different age groups, the MF prevalence was lowest in 1–9 years (11.05%, 95% *CI*: 9.80–12.43%), increased sharply to 50.57% (95% *CI*: 47.87–53.26%) in 10–19 years, peaked at 82.31% (95% *CI*: 79.39–84.90%) in 40–49 years, and then dropped to 76.17% (95% *CI*: 72.84–79.20%) in 60 years and aobve.

**Table 3:** Crude onchocerciasis MF prevalence and density in Sierra Leone after five annual rounds of MDA

	Baseline survey				2010 Epidemiological Evaluation				Percentage reduction (%)			Significance test (*P*-values)		
	No. examined	MF prev (%)	AMD-all (mf/snip)	AMD-positive (mf/snip)	No. examined	MF prevalence (95% *CI*)	AMD-all (mf/snip) (95% *CI*)	AMD-positive (mf/snip) (95% *CI*)	MF prev	AMD-all	AMD-positive	MF prev	AMD-all	AMD-positive
Overall	7116	53.09	15.33	28.87	5621	21.12 (20.07–22.20)	1.75 (1.48–2.02)	8.29 (7.07–9.50)	60.22	88.58	71.29	0	0	0
*By district*														
Bo	1499	54.3	18.91	34.82	1079	25.77 (23.24–28.46)	2.95 (2.11–3.80)	11.46 (8.40–14.53)	52.55	84.4	67.09	0	0	0
Bombali	368	57.88	16.85	29.1	494	16.19 (13.21–19.70)	1.58 (0.86–2.29)	9.72 (5.71–13.72)	72.02	90.62	66.6	0	0	0
Bonthe	180	40.56	7	17.25	165	26.67 (20.51–33.16)	3.05 (0.41–5.68)	11.42 (1.71–21.13)	34.25	56.43	33.8	0.009	0.004	0.247
Kailahun	183	49.73	12.06	24.25	118	23.73 (16.96–32.16)	2.01 (0.47–3.55)	8.46 (2.31–14.62)	52.28	83.33	65.11	0	0	0
Kambia	155	61.94	17.8	28.73	98	23.47 (16.18–32.76)	1.04 (0.43–1.65)	4.41 (2.24–6.58)	62.11	94.16	84.65	0	0	0
Kenema	286	45.12	6.51	14.42	101	20.79 (14.02–29.70)	1.35 (0.18–2.51)	6.48 (1.09–11.86)	53.9	79.26	55.06	0	0	0.217
Koinadugu	168	58.33	18.38	31.51	58	6.90 (2.71–16.43)	0.05 (0.00–0.11)	0.75 (0.00–1.55)	88.18	99.73	97.62	0	0	0.005
Kono	1105	39.01	14.63	37.48	521	9.02 (6.85–11.79)	0.32 (0.18–0.46)	3.53 (2.31–4.75)	76.87	97.81	90.58	0	0	0
Moyamba	1055	58.2	12.06	20.74	988	29.96 (27.19–32.89)	1.64 (1.27–2.01)	5.46 (4.35–6.58)	48.52	86.4	73.67	0	0	0
Port Loko	653	57.12	13.83	24.21	738	26.02 (22.98–29.30)	3.10 (1.80–4.40)	11.91 (7.12–16.71)	54.45	77.58	50.81	0	0	0
Pujehun	560	53.57	9.84	18.36	348	7.76 (5.39–11.03)	0.60 (0.06–1.14)	7.76 (1.03–14.49)	85.52	93.9	57.73	0	0	0.002
Tonkolili	904	60.4	22.09	36.5	913	16.10 (13.86–18.63)	0.67 (0.43–0.91)	4.16 (2.81–5.50)	73.34	96.97	88.6	0	0	0
*By sex*														
Male	3461	55.19	21.11	38.16	2805	24.49 (22.94–26.12)	2.55 (2.04–3.06)	10.40 (8.43–12.36)	54.24	87.92	72.75	0	0	0
Female	3655	51.11	9.94	19.38	2816	17.76 (16.39–19.21)	0.96 (0.77–1.15)	5.40 (4.40–6.40)	64.12	90.34	72.14	0	0	0
*Age groups*														
1–9	2182	11.05	0.91	8.24	1930	1.71 (1.22–2.39)	0.04 (0.02–0.06)	2.47 (1.49–3.45)	82.55	95.6	70.02	0	0.168	0.02
10–19	1317	50.57	6.91	13.67	889	16.09 (13.82–18.65)	0.80 (0.51–1.08)	4.94 (3.31–6.58)	66.4	88.42	63.86	0	0	0
20–29	780	78.21	19.94	25.5	648	38.27 (34.61–42.07)	3.68 (2.48–4.88)	9.58 (6.58–12.58)	49.09	81.54	62.43	0	0	0
30–39	824	80.95	28.88	35.64	624	37.18 (33.48–41.04)	3.49 (2.34–4.64)	9.38 (6.43–12.33)	52.41	87.92	73.68	0	0	0
40–49	735	82.31	35.94	43.66	542	38.75 (34.74–42.91)	4.09 (2.65–5.54)	10.56 (6.99–14.13)	51.2	88.62	75.81	0	0	0
50–59	575	81.39	30.37	37.31	416	37.02 (32.52–41.76)	2.61 (1.15–4.06)	7.04 (3.18–10.89)	52.55	91.41	81.13	0	0	0
≥ 60	684	76.17	21.58	28.34	566	29.33 (25.73–33.21)	2.10 (1.47–2.73)	7.17 (5.22–9.12)	59.74	90.27	74.7	0	0	0

#### MF density

Among all 7116 people tested, the overall arithmetic mean density (AMD-all) of MF was 15.33 microfilariae (mf)/snip (95% *CI*: 14.43–16.24 mf/snip), 21.11 mf/snip (95% *CI*: 19.49–22.72 mf/snip) in males and 9.94 mf/snip (95% *CI*: 9.09–10.79 mf/snip) in females (*P* < 0.001). AMD-all ranged from 6.51 mf/snip (95% *CI*: 3.47–9.54 mf/snip) in Kenema district to 22.09 mf/snip (95% *CI*: 19.28–24.91 mf/snip) in Tonkolili district. Among the 3778 people that tested MF positive, the arithmetic mean density (AMD-positive) was 28.87 mf/snip (95% *CI*: 27.29–30.46 mf/snip), 38.16 mf/snip (95% *CI*: 35.47–40.84 mf/snip) in males and 19.38 mf/snip (95% *CI*: 17.85–20.92 mf/snip) in females (*P* < 0.001). The AMD-positive ranged from 14.42 mf/snip (95% *CI*: 7.91–20.94 mf/snip) in Kenema district to 37.48 mf/snip (95% *CI*: 31.36–43.60 mf/snip) in Kono district. There was no trend observed for MF prevalence between the regions for the AMD-all and AMD-positive.

The AMD-all was lowest among the age group of 1–9 years (0.91 mf/snip, 95% *CI*: 0.51–1.30 mf/snip), increased sharply to 6.91 mf/snip (95% *CI*: 5.92–7.91 mf/snip) in the age group of 10–19 years, peaked at 35.94 mf/snip (95% *CI*: 31.44–40.44 mf/snip) in the age group of 40–49 years, and then dropped to 21.58 mf/snip (95% *CI*: 18.44–24.72 mf/snip) in the age group of ≥60 years. The AMD-positive followed the same pattern as AMD-all (Table 3).

### Onchocerciasis situation in 2010

#### MF prevalence

In 2010, a total of 5621 people were tested in all the 39 villages: 2805 (49.9%) males and 2816 (50.1%) females. The overall MF prevalence was 21.12% (95% *CI*: 20.07–22.20%): 24.49% (95% *CI*: 22.94–26.12%) in males and 17.76% (95% *CI*: 16.39–19.21%) in females. Point MF prevalence at each sentinel site showed a general trend of decline in prevalence across the country from the baseline, which was more evident in the northern part of the country (Fig. 2). Compared with the baseline, overall MF prevalence dropped by 60.22% (*P* < 0.001): 54.24% among males (*P* < 0.001) and 64.12% among females (*P* < 0.001). The MF prevalence among districts ranged from 6.90% (95% *CI*: 2.71–16.43%) in Koinadugu district to 29.96% (95% *CI*: 27.19–32.89%) in Moyamba district. Reduction in MF prevalence among districts was, in general, greater than 50% (*P* < 0.05), except for Bonthe and Moyamba districts which had 34.25% (*P* < 0.01) and 48.52% (*P* < 0.001) reductions in prevalence respectively. Reductions in MF prevalence in Koinadugu and Pujehun districts were both over 80% (*P* < 0.001). For the age groups, the MF prevalence showed similar age pattern as at the baseline but was at a significantly reduced level (Table 3). Among the age groups, the highest reduction in MF prevalence (82.55%) was recorded in 1–9 years followed by the reduction in 10–19 years (66.40%).

#### MF density

The overall AMD-all was 1.75 mf/snip (95% *CI*: 1.48–2.02 mf/snip) and the AMD-positive was 8.29 mf/snip (95% *CI*: 7.07–9.50 mf/snip), a significant drop of 88.58% (*P* < 0.001) and 71.29% (*P* < 0.001) from the baseline, respectively. Compared to the baseline, the AMD-all was 2.55 mf/snip (95% *CI*: 2.04–3.06 mf/snip) in males, a decrease of 87.92% (*P* < 0.001), and 0.96 mf/snip (95% *CI*: 0.77–1.15 mf/snip) in females, a decrease of 90.34% (*P* < 0.001). The AMD-positive was 10.40 mf/snip (95% *CI*: 8.43–12.36 mf/snip) in males, a decrease of 72.75% (*P* < 0.001) and 5.40 mf/snip (95% *CI*: 4.40–6.40mf/snip) in females, a decrease of 72.14% (*P* < 0.001). There was a significant difference in AMD between males and females (*P* < 0.05). Among districts, the AMD-all ranged from 0.05 mf/snip (95% *CI*: 0.00–0.11 mf/snip) in Koinadugu district to 3.10 mf/snip (95% *CI*: 1.80–4.40 mf/snip) in Port Loko district, in general a significant reduction of > 70% (*P* < 0.05) across districts, with the exception of Bonthe district, which showed a reduction of 56.43% (*P* < 0.01). The AMD-positive ranged from 0.75 mf/snip (95% *CI*: 0.00–1.55 mf/snip) in Koinadugu district to 11.91 mf/snip (95% *CI*: 7.12–16.71 mf/snip) in Port Loko district, in general a significant reduction of > 50% (*P* < 0.05), with the exception of Bonthe district where the reduction was 33.80% (*P* > 0.05). Among the age groups, the AMD-all and AMD-positive showed similar age distribution pattern as the baseline, however, there was in general > 80% reduction for AMD-all and > 60% reduction for AMD-positive (Table 3).

## Discussion

The following distinct epidemiological patterns were determined in the MF prevalence from the survey data: 1) higher in males than in females, 2) lowest in 1–9 years followed by that in 10–19 years, and 3) higher in the northern districts than in the southern and eastern districts. Distinct epidemiological patterns were also noted in the MF density. AMD-all or AMD-positive was higher in males than in females, was lowest among the age group of 1–9 years followed by that in the age group of 10–19 years, and was highest among the age group of 40–49 years.

In general, males had higher *O. volvulus* infection levels than females. Although MF prevalence in males was only slightly higher than in females, the MF density for all surveyed and positive-only participants was about twice as high in males as in females. The similar male/female differences in onchocerciasis prevalence, density of infection and clinical disease have been discussed by many authors [27–33]. However, it should be noted that Gbakima and Sahr detected no difference in *O. volvulus* infection between males and females in their Sierra Leone study [34]. It has been suggested that higher *O. volvulus* infection in males, compared to females, may be due to increased exposure to black flies among males through fishing and other activities that take place in close proximity to rivers [29, 35, 36].

Lower age groups (1–9 years and 10–19 years) had the lowest MF prevalence and density even within highly endemic areas. MF prevalence and density were highest in the age group of 40–49 years. This age trend was similar to the results of a study in Nigeria that found *O. volvulus* MF prevalence and intensity increasing with age [28]. This may be explained by the fact that adults with growing age have more exposure to the bite of infected black flies. It is reported that maternal *O. volvulus* infection can be transmitted, even in utero, to their children and that children of onchocerciasis-infected mothers are more likely to be infected with onchocerciasis [37, 38]. The fact that females in our surveyed villages were less infected than men suggests that children in these villages may have had less exposure to the bite of infected black flies or in utero transmission.

It has been suggested that the regional and district variations of onchocerciasis prevalence at baseline could be linked with vector ecology and density, which also depend on the distance of villages studied from the river basin and the geography of the river [39]. Previous studies in Sierra Leone showed that *O. volvulus* MF prevalence was higher in the forest (71.8%) than in Savannah villages (51.9%) [30]. In general, the eastern region has the forest strain of the *O. volvulus* parasite, characterised by low intensity of infection, mild skin disease and relatively low blindness rate; the southern region has a mixture of forest and Savannah strains of the parasite, characterised by high infection intensity, mild skin disease and relatively higher blindness rate (sometimes higher than blindness rates recorded in the Savannah area); and the northern region has the Savannah strain of the parasite, characterised by high infection intensity, mild skin disease, and relatively high blindness rate [14, 34]. Our survey data may have reflected these.

The 2010 evaluation revealed a significant decrease in onchocerciasis MF prevalence and density in the same 39 sentinel villages after just five rounds of annual MDA. The same epidemiological dynamics as the baseline was observed in the 2010 evaluations, i.e. MF prevalence was higher in males than in females and MF density was twice as high in males as in females. The regional disease distribution observed at baseline (the northern districts with relatively higher prevalence, followed by the southern and then the eastern having the lowest relative prevalence) appeared to have changed significantly after five years of MDA. In 2010, the northern districts had the lowest average MF prevalence relative to the eastern and southern districts. This observation could not be explained by differences in treatment coverage (similar in all the regions) but may be due to the responsiveness of different parasite strains to treatment and further studies are needed to clarify this. The significant reduction in *O. volvulus* infection after five years of MDA is similar to the results from other countries. In Cameroon, onchocerciasis MF prevalence in the Fundong health district decreased from 60.0% to 3.5% after six rounds of continuous MDA using the CDTI strategy [40]. After seven years (1995–2001) of ivermectin treatment in three endemic villages of the Etung Local Government Area of Lower Cross River Basin, Nigeria, MF prevalence decreased from 63.3% at baseline to 39.3% and community MF density dropped from 7.11 to 2.31 mf/snip [38].

Significant progress has been made on onchocerciasis elimination in Sierra Leone. Effective epidemiological and programme coverage in all endemic areas was reached and has been maintained since 2005. By 2017, considering the missed MDA due to Ebola virus disease in 2014, a total of 10 rounds of effective MDA have been completed. Per the current WHO recommendations, 12–15 years of MDA are needed for onchocerciasis elimination [22]. It is anticipated that additional 2–5 annual rounds of MDA may bring Sierra Leone to the point of stopping MDA for onchocerciasis. However, there are currently no guidelines on the prevalence threshold and methodology for deciding when to move to stop-MDA evaluation. While continuing with the ivermectin MDA in the 12 districts, the NTDP has established a national onchocerciasis elimination committee (NOEC), per WHO recommendations, which will review the programme progress and recommend a way forward for the NTDP on onchocerciasis elimination.

There are certain limitations in this study. First, the baseline data were obtained over a long period. Some villages were studied pre-war in 1988–1990 and others were studied post-war in 2002–2004 with a gap of about 11 years. It is possible that the epidemiological situation may have changed in villages studied in the earlier years. This is exemplified by the fact that 7 of the originally selected villages could not be traced. Therefore, comparing with the baseline data collected pre-war, the reduction in infection seen in 2010 may have not been entirely due to the impact of MDA. However, we tried to minimize this effect by including more villages with more recent data (31 sites with data from 2002 to 2004 and eight sites with data from 1998 to 1990). Villages with older data (1988–1990) were only selected when more recent data (2002–04) were not available for a district. Second, in the 2010 evaluation, only persons aged five years and above were studied while all persons aged over one year old were studied at baseline. This resulted in fewer children aged 1–9 years examined in the 2010 evaluation. The decision not to study children below five years of age was based on the high refusal rate to skin snip in this age group observed in communities during the baseline studies. Newly developed serological tools (OV16 rapid diagnostic tests [RDTs] and OV16 ELISA) should be considered for better compliance in future evaluations [41–43]. Third, the sentinel sites surveyed in 2010 did not include hypo-endemic villages. As the focus for onchocerciasis has shifted from control to elimination and hypo-endemic villages have been treated through LF MDA. It is essential to include hypo-endemic villages in future evaluations to have a full picture of the current onchocerciasis for national programme decision-making.

## Conclusions

There was a significant reduction of onchocerciasis MF prevalence and MF density across the 12 rural onchocerciasis-endemic districts of Sierra Leone after five annual MDAs. The results suggested that the onchocerciasis elimination programme in Sierra Leone was on course to reach the objective of eliminating onchocerciasis in Sierra Leone by the year 2025. However, MDA needs to continue in all 12 districts with required treatment coverage to reach the goal of interrupting transmission. Further evaluations across the country are needed to assist the NTDP with programme decision-making.

## Additional file

Additional file 1: Multilingual abstracts in the five official working languages of the United Nations. (PDF 483 kb)

## Abbreviations

AMD-all: Arithmetic mean density for all persons tested
AMD-positive: Arithmetic mean density among people that tested MF positive
APOC: African Programme for Onchocerciasis Control
CDD: Community-directed drug distributor
CDTI: Community-Directed Treatment with Ivermectin
*CI*: Confidence interval
DHMT: District Health Management Team
LF: Lymphatic filariasis
MDA: Mass drug administration
mf: Microfilariae
MF: Microfilaridermia
MOHS: Ministry of Health and Sanitation
NOCP: National Onchocerciasis Control Program
NOEC: National Onchocerciasis Elimination Committee
NTD: Neglected tropical diseases
NTDP: Neglected Tropical Disease Program
OCP: Onchocerciasis Control Programme in West Africa
RDT: Rapid diagnostic test
SIZ: Special Intervention Zones
USAID: United States Agency for International Development
WHO: World Health Organisation
