# Supplementary material for: Impact of five annual rounds of mass drug administration with ivermectin on onchocerciasis in Sierra Leone
Source: Infect Dis Poverty. 2018 Apr 6;7:30. doi: 10.1186/s40249-018-0410-y (PMC5890354; doi:10.1186/s40249-018-0410-y)
Supplement: Supplementary file 1 — Multilingual abstracts in the five official working languages of the United Nations. (PDF 483 kb) [file 40249_2018_410_MOESM1_ESM.pdf]

Translation of the abstract into the five official working languages of the United Nations

تأثير خمس جولات سنوية للإدارة الجماعية للأدوية باستخدام دواء إيفيرمكتين على داء كُلابية الذَّنَب في سيراليون

جوزيف بي. كوروما، سانتيجي سيساي، عبدول كونتيه، بنيامين كودو، جوسوفو باي، محمد باه، مصطفى سوني، ماري هودجز، يوبي تشانغ، موسى جي. بوكاري

نبذة مختصرة

معلومات عامة: يستوطن داء كُلابية الذَّنَب في 12 مقاطعة من أصل 14 مقاطعة صحية في سيراليون. وقد تحققت تغطية علاجية جيدة للدواء الموجه للمجتمع باستخدام دواء الإيفيرمكتين بين عامي 2005 و2009 بعد الحرب الأهلية التي استمرت 11 سنة. وقد أُجريت دراسات استقصائية في مواقع رصد الأمراض المُعدية لتقييم تأثير خمس جولات سنوية لتوزيع دواء الإيفيرمكتين.

النظريات: بشكل إجمالي، تمت معاينة 39 قرية لرصد الأمراض المُعدية من المناطق شديدة ومتوسطة الاستيطان للأمراض عبر 12 مقاطعة مستوطنة للمرض باستخدام العينات الجلدية في عام 2010. وتم تحليل النتائج ومقارنتها مع البيانات الأساسية المستمدة من نفس القرى البالغ عددها 39 قرية.

النتائج: بلغ معدل انتشار المكروفيلارية (MF) في الـ 39 قرية المُخصصة لرصد الأمراض المُعدية 53.10% من المقياس الأساسي. كان معدل انتشار (MF) أعلى في الفئات العمرية الأكبر سنًا، حيث كان أدنى معدل في الفئة العمرية من سنة واحدة إلى 9 سنوات (11.00%)، وكان أعلى معدل في الفئة العمرية من 40 إلى 49 سنة (82.31%). كان معدل انتشار (MF) أعلى في الفئات العمرية الأكبر سنًا، حيث كان أدنى معدل في الفئة العمرية من سنة واحدة إلى 9 سنوات (11.00%)، وكان أعلى معدل في الفئة العمرية من 40 إلى 49 سنة (82.31%). ولقد كان انتشار (MF) وكثافته أعلى بين الذكور من الإناث. في عام 2010، وبعد خمس جولات من الإدارة الجماعية للأدوية، انخفض المعدل العام لانتشار (MF) بنسبة 60.26% من 53.10% إلى 21.10%؛ انخفض المتوسط العام لكثافة (MF) بين النتائج الإيجابية بنسبة 71.29% من 28.87 mf/snip إلى 8.29 mf/snip؛ وانخفض المتوسط العام لكثافة (MF) لدى جميع الأشخاص الذين تم فحصهم بنسبة 88.58% من 15.33 mf/snip إلى 1.75 mf/snip. وقد انخفض انتشار (MF) بنسبة 50% في عشرة مقاطعات من أصل 12 مقاطعة مستوطنة للمرض. وقد انخفضت كثافة (MF) بنسبة 50% في المتوسط بين النتائج الإيجابية في إحدى عشرة مقاطعة من أصل 12 مقاطعة.

الاستنتاجات: تم تسجيل انخفاض كبير في الانتشار ومتوسط كثافة (MF) لداء كُلابية الذَّنَب في جميع المقاطعات الاثنا عشرة في سيراليون بعد خمس جولات سنوية للإدارة الجماعية للأدوية مع تغطية علاجية فعالة. أشارت النتائج إلى أن برنامج القضاء علي داء كُلابية الذَّنَب في سيراليون كان في طريقه إلى بلوغ هدف القضاء علي داء كُلابية الذَّنَب في البلاد بحلول عام 2025. ينبغي أن تستمر هذه الجولة السنوية للإدارة الجماعية للأدوية باستخدام دواء إيفيرمكتين في جميع المقاطعات الاثنا عشرة، وهناك حاجة إلى إجراء المزيد من التقييمات في جميع أنحاء البلاد لمساعدة برنامج أمراض المناطق المدارية المُهملة (NTDP) علي اتخاذ القرارات بشأن البرنامج.

Translated from English version into Arabic by Nader Hassan and Ibraheem Al-Luaibi, through

TRANSLATORS
WITHOUT BORDERS

五年五次的大规模伊维菌素治疗对塞拉利昂盘尾丝虫病的控制效果

Joseph B. Koroma, Santigie Sesay, Abdul Conteh, Benjamin Koudou, Jusufu Paye, Mohamed Bah, Mustapha Sonnie, Mary H. Hodges, Yaobi Zhang, Moses J. Bockarie

摘要

引言 :盘尾丝虫病流行于塞拉利昂 14 个卫生区中的 12 个。经历了 11 年的内战之后，在 2005 年至 2009 年期间，社区主导的伊维菌素治疗覆盖率达到了良好的水平。2010 年进行了哨点现场调查，以评估五年来每年一次伊维菌素治疗的效果。
方法 : 2010 年对全国 12 个流行卫生区高、中度流行地区的 39 个哨点村进行了皮肤切样调查，将结果同该 39 个村的基线资料进行了分析比较。
结果 : 39 个哨点村平均基线微丝蚴皮症（MF）患病率为 53.10%。MF 发病率在年长者中较高，其中 1 - 9 岁年龄组最低（11.00%），而 40 - 49 岁年龄组最高（82.31%）。MF 阳性者中平均微丝蚴密度为每切样 28.87 个（mf/ snip）。微丝蚴密度随年龄而增加，其中 1 - 9 岁年龄组最低，40 - 49 岁年龄组最高。男性 MF 患病率和密度均高于女性。2010 年经过五轮每年一次的大规模伊维菌素治疗后，总体 MF 患病率由 53.10%下降到 21.10%，下降了 60.26%。阳性者中平均 MF 密度从 28.87 mf / snip 降至 8.29 mf / snip，下降了 71.29%。所有被调查者中平均 MF 密度从 15.33 mf / snip 降至 1.75 mf / snip，下降了 88.58%。在 12 个流行卫生区中，MF 患病率下降了 50%以上的有 10 个，阳性者平均 MF 密度降低了 50%以上的有 11 个。
结论 : 经过 5 年有效治疗覆盖率的大规模伊维菌素治疗后，所有 12 个塞拉利昂流行卫生区的盘尾丝虫病患病率和平均 MF 密度均有显着下降。结果表明，塞拉利昂的盘尾丝虫病国家项目正在向实现到 2025 年消除国内盘尾丝虫病的目标迈进。现阶段，所有 12 个流行区应继续推行伊维菌素大规模治疗，必要时，需要在全国范围内进行进一步的评估以协助国家项目做出方案决策。

Translated from English version into Chinese by Yao-Bi Zhang

Impact de cinq tournée d'administration de masse d'ivermectine sur l'onchocercose au Sierra Leone

Joseph B. Koroma, Santigie Sesay, Abdul Conteh, Benjamin Koudou, Jusufu Paye, Mohamed Bah, Mustapha Sonnie, Mary H. Hodges, Yaobi Zhang, Moses J. Bockarie

RÉSUMÉ

Contexte : L'onchocercose est endémique dans 12 des 14 districts sanitaires du Sierra Leone. Une bonne couverture de traitement a été obtenue grâce aux distributions d'ivermectine aux communautés entre 2005 et 2009, au lendemain d'une guerre civile qui a duré onze ans. Des relevés sur sites sentinelles ont été réalisés en 2010 afin d'évaluer l'impact de cinq tournées annuelles de distribution d'ivermectine.
Méthodes : Au total, 39 villages sentinelles situés dans des zones d'hyper- et mésoendémie des 12 districts où l'onchocercose est endémique ont été surveillés au moyen de biopsies cutanées exsangues en 2010. Les résultats de ces prélèvements ont été analysés et comparés aux données initiales des 39 mêmes villages.
Résultats : La prévalence moyenne de la microfilarémie dans les 39 villages sentinelles était de 53,10 % au début de l'étude. Elle était plus élevée dans les groupes d'âge plus avancé, avec un minimum dans le groupe de 1 à 9 ans (11,00 %) et un maximum dans le groupe de 40 à 49 ans (82,31 %). La densité totale moyenne de la microfilarémie parmi les sujets positifs était de 28,87 microfilaires par biopsie, avec un minimum dans le groupe de 1 à 9 ans et une augmentation avec

l’âge jusqu’au maximum dans le groupe de 40 à 49 ans. La prévalence et la densité de la microfilarémie étaient plus élevées chez les sujets de sexe masculin. En 2010, après cinq tournées d’administration de masse de médicaments, la prévalence totale de la microfilarémie a baissé de 60,26 %, de 53,10 % à 21,10 % ; la densité totale moyenne de microfilaires chez les sujets positifs a baissé de 71,29 %, de 28,87 à 8,29 microfilaires par biopsie, et la densité totale moyenne des microfilaires parmi tous les sujets examinés a baissé de 88,58 % , de 15,33 à 1,75 microfilaires par biopsie. Dix des douze districts d’onchocercose endémique ont connu une baisse de plus de 50 % de la prévalence de la microfilarémie. Onze de ces douze districts présentaient une baisse ≥ 50 % de la densité moyenne de microfilaires chez les sujets positifs.
Conclusions : Une baisse significative de la prévalence et de la densité moyenne des microfilaires d’onchocerques a été relevée dans les 12 districts du Sierra Leone après cinq distributions de masse de médicaments, qui ont permis une couverture efficace par le traitement. Les résultats suggèrent que le programme d’élimination de l’onchocercose au Sierra Leone est bien parti pour atteindre l’objectif d’élimination de la maladie dans le pays d’ici 2025. Les distributions de masse d’ivermectine annuelles devront être poursuivies dans les douze districts. De nouvelles évaluations sont nécessaires dans tout le pays, afin d’aider le programme national contre les maladies non transmissibles (NTDP) à prendre ses décisions programmatiques.

Translated from English version into French by Suzanne Assenat, through

TRANSLATORS
WITHOUT BORDERS

Влияние на онхоцеркоз пяти ежегодных циклов массового применения ивермектина в Сьерра-Леоне

Джозеф Б. Корома, Сантиги Сесай, Абдул Конте, Бенджамин Куду, Джусуфу Пайе, Мохамед Ба, Мустафа Сонни, Мэри Х. Ходжес, Яоби Чжан, Моузес Дж. Бокари

АННОТАЦИЯ

Справочная информация: Онхоцеркоз эндемичен в 12 из 14 округов здравоохранения Сьерра-Леоне. Эффективный охват лечением ивермектином на базе общины был достигнут с 2005 до 2009 года после 11-летнего гражданского конфликта.
Методы: Всего в 2010 году было обследовано 39 дозорных деревень из гипер- и мезоэндемичных районов в 12 эндемичных районах на основе исследования срезов кожи. Результаты были проанализированы и сопоставлены с исходными данными из тех же 39 деревень.
Результаты: Средняя распространенность микрофилярдемии среди 39 дозорных деревень на исходном уровне составляла 53,10%. Распространенность микрофилярдемии была выше у старших возрастных групп, причем самая низкая в возрастной группе 1 - 9 лет (11,00%) и самая высокая в возрастной группе 40-49 лет (82,31%). Общая средняя плотность микрофилядермии среди инфицированных составляла 28,87 микрофилярий (мф)/срез,

увеличиваясь с возрастом с самым низким показателем в возрастной группе 1 - 9 лет и самым высоким в возрастной группе 40-49 лет. Среди мужчин наблюдалась более высокая распространенность и плотность микрофилядермии, чем среди женщин. В 2010 году, после пяти циклов массового применения лекарственных препаратов, общая распространенность микрофилядермии снизилась на 60,26% с 53,10% до 21,10%; общая средняя плотность микрофилядермии среди инфицированных уменьшилась на 71,29% с 28,87 мф/срез до 8,29 мф/срез; и общая средняя плотность микрофилядермии среди всех обследованных лиц снизилась на 88,58% с 15,33 мф/срез до 1,75 мф/срез. В десяти из 12 эндемичных районов наблюдалось снижение на >50% распространённости микрофилядермии. В одиннадцати из 12 районов отмечено снижение на ≥ 50% средней плотности микрофилядермии среди инфицированных.
Итоги: Значительное снижение распространённости и средней плотности онхоцеркоза было зарегистрировано во всех 12 районах Сьерра-Леоне после пяти ежегодных циклов массового применения лекарства с эффективным охватом лечения. Результаты показали, что программа ликвидации онхоцеркоза в Сьерра-Леоне направлена на достижение цели ликвидации онхоцеркоза в стране к 2025 году. Ежегодное массовое применение лекарств с ивермектином должно продолжаться во всех 12 районах, и необходимы дальнейшие оценки по всей стране, чтобы помочь Программе по забытым тропическим болезням в принятии решений.

Translated from English version into Russian by Oksana Rozhko and Liudmila Tomanek, through

TRANSLATORS
WITHOUT BORDERS

Repercusión de la administración de las cinco series anuales del conjunto de medicamentos con ivermectina contra la oncocercosis en Sierra Leona

Joseph B. Koroma, Santigie Sesay, Abdul Conteh, Benjamin Koudou, Jusufu Paye, Mohamed Bah, Mustapha Sonnie, Mary H. Hodges, Yaobi Zhang, Moses J. Bockarie

RESUMEN

Contexto: la oncocercosis es endémica en 12 de los 14 distritos sanitarios de Sierra Leona. Entre 2005 y 2009, tras 11 años de conflicto civil, se consiguió una buena cobertura del tratamiento con ivermectina en la población. En 2010 se llevaron a cabo estudios de vigilancia para valorar la repercusión de las cinco series anuales de distribución de ivermectina.
Métodos: en 2010 se estudiaron un total de 39 aldeas vigiladas, situadas en zonas hiperendémicas o mesoendémicas a lo largo de los 12 distritos endémicos, mediante la utilización de muestras de piel. Los resultados se analizaron y compararon con los datos de referencia de las mismas 39 aldeas.
Resultados: la media de prevalencia de la microfilaridermia (MF) a lo largo de las 39 aldeas era del 53,10 % según los datos de referencia. La prevalencia de la MF era mayor en los grupos de mayor edad. La menor fue la del grupo de edad entre 1 y 9 años (11,00 %) y la mayor en el grupo de 40 a 49 años (82,31 %). La densidad media general de la MF entre los positivos era de 28,87 microfilarias

(mf) por muestra, aumentando con la edad, con la menor tasa entre el grupo de 1 a 9 años y la mayor en el grupo de 40 a 49 años. Los varones tuvieron una mayor prevalencia y densidad de MF que las mujeres. En 2010, tras la administración de las cinco series del conjunto de medicamentos, la prevalencia general de la MF disminuyó en el 60,26 % de los casos de un 53,10 % a un 21,10 %, la densidad media general de la MF entre los positivos disminuyó en el 71,29 % de los casos de 28,87 a 8,29 mf por muestra y la densidad media general de la MF entre todas las personas examinadas disminuyó en el 88,58 % de los casos de 15,33 a 1,75 mf por muestra. Diez de los doce distritos tuvieron una reducción de la prevalencia de la MF > al 50 %. Once de los doce distritos tuvieron una reducción ≥ al 50 % en la densidad media de la MF entre los positivos.

Conclusión: se registró una reducción de la prevalencia y densidad media de la MF por oncocercosis en los 12 distritos de Sierra Leona tras la administración de las cinco series anuales del conjunto de medicamentos con cobertura efectiva del tratamiento. Los resultados han indicado que el programa en curso de eliminación de la oncocercosis en Sierra Leona tiene como objetivo erradicarla del país para el año 2025. La administración anual del conjunto de medicamentos con ivermectina debería continuar en los 12 distritos y se necesitan más estudios a lo largo del país para ayudar al Programa de Enfermedades Tropicales Desatendidas (NTDP según sus siglas en inglés) en la toma de decisiones del programa.

Translated from English version into Spanish by Patricia Cassoni and Fran Egea, through

TRANSLATORS
WITHOUT BORDERS
